# The shifts in the structure of the prokaryotic community of mountain-grassland soil under the influence of artificial larch plantations

**DOI:** 10.1371/journal.pone.0263135

**Published:** 2022-02-18

**Authors:** Ekaterina Ivanova, Evgeny Abakumov, Nadezda Vasilyeva, Alexei Zverev, Artem Vladimirov, Natalia Ksenofontova, Evgeny Andronov, Igor Kostenko

**Affiliations:** 1 Dokuchaev Soil Science Institute, Moscow, Russia; 2 Agrophysical Research Institute, St. Petersburg, Russia; 3 St. Petersburg State University, St. Petersburg, Russia; 4 All-Russian Research Institute of Agricultural Biology, Pushkin, St. Petersburg, Russia; 5 Nikitsky Botanical Garden–National Scientific Centre of RAS, Yalta, Russia; City University of New York, UNITED STATES

## Abstract

Creation of artificial forest plantations on a global scale is one of the ways to mitigate the negative effects of climate change on ecosystems, at the same time providing soil protection from erosion, regulation of the hydrological regime and carbon sequestration in soils of different natural and climatic zones. However, the change of the dominant plant community cause significant ecosystem changes, reflecting at the structure and functioning of the soil microbial complex as well. The shifts in prokaryotic community of the meadow soil resulting from the conversion of the native meadow (further grassland) phytocenosis to the artificial forest plantations was investigated with the use of NGS sequencing technology and metabarcoding approach–amplicon sequencing of V4 region of 16 S rRNA (performed on Illumina Miseq platform). The identified shifts in taxonomic structure and diversity may be the result of changes in the physic-chemical conditions of soils and, on the other hand, may serve as indicators of such changes. Cultivation of larch led to an increase in the diversity of the prokaryotic community and its stratification by depth. The acidifying effect of larch manifested itself in an increase in the proportion and diversity of acidobacteria, in the abundance of oligotrophic microorganisms of phyla *Chloroflexi*, *Firmicutes*, and a simultaneous comparative decrease in the bacteria of *Verrucomicrobia* phylum, alphaproteobacteria of or. *Rhizobiales* and *Burkholderiales*. The absence of clearly expressed dominants in the prokaryotic community, as well as a significant increase in alpha-diversity indices, compared with the control plot of native mountain-meadow soil under grassland vegetation, suggests a transitional nature of the soil ecosystem of artificial forest plantations.

## Introduction

Creation of man-made forests is one of the ways to mitigate the negative effects of climate change on ecosystems, while ensuring soil protection against erosion, regulation of the hydrological regime of soils, carbon sequestration, etc. Among the man-made forests created in the second half of the 20th century on the Crimean plateau with a total area of about 3 thousand hectares, coniferous (pine) forests dominate, occupying up to 70% of forested areas [1]. The main areas of man-made forests are located on the Ai-Petri yaila, where, in addition to Scot’s pine (*Pinus sylvestris* L.), forests of silver birch (*Betula pendula* Roth), aspen (*Populus tremula* L.) and plane-tree maple (*Acer pseudoplatanus* L.) are wide-spread. Small areas of nutwood (Corylus avellana L.), wild pear tree (*Pyrus pyraster* Burgsd), spruce (*Picea abies* (L.) H. Karst.) and one area of Siberian larch (*Larix sibirica* Ledeb), with an area of about 0.7 hectares, present here as well. At the beginning of the last century M.E. Tkachenko [2] drew attention to the strong acidifying effect of larch stands, but nevertheless he believed that, in general, this species has a positive effect on forest soils, improving the structure and contributing to the accumulation of mobile forms of nitrogen and phosphorus. The data of F.I. Khakimov et al. [3] also indicate a higher acidity of soils under 100-year-old larch stands and a greater amount of silica powdering in the upper soil layer compared to soil under a mixed forest. The conversion of secondary forest into larch cultivation led to changes in the qualitative and quantitative composition of soil organic matter (SOM), which was expressed in an increase in its light fraction, and, accordingly, a decrease in the amount of stable SOM in the soils of northeastern China [4]. This was accompanied by a simultaneous decrease in the values of microbiological parameters–the amount of microbial biomass, enzymatic activity, and C and N mineralization rate [4, 5]. However, the study of Crimean soils showed that larch growth contributed to an overall increase in humus content in the upper part of the profile, compared with mixed forest and long-term fallow lands [3, 6].

The plant-soil-microorganism system is a key research issue in a wide range of scientific disciplines. Microorganisms, which are directly or indirectly associated with the plant community, quickly respond to changes in soils, and can act as a sensitive indicator of both changes in ecological conditions and the way of chemical and soil-biological processes in soil. The latter became possible mainly due to the application of meta-omics approaches to the study of soil microbiomes, as well as high-throughput new generation sequencing technologies, which allowed to assess the close-to-real scale of the diversity and taxonomic composition of environmental microbiomes.

Shifts in the microbial community as a result of changing plant communities are part of a broad research objective in considering plants as an ecological driver of soil microbiocenosis. Traditionally such influences are linked to differences in the spectrum of root exudates of different plants, but in the case of an abrupt change in phytocoenosis, in particular a change in the herbaceous community by a forest biocoenosis, structural changes in the soil itself will also play a major role. Recent studies [6] have shown a significant transformative effect of artificial forest plantations on the soil ecosystem. The structural state and aggregate composition of soils, humus content, acidity, and iron content of organic-mineral compounds changed under the influence of tree species cultivation compared to soils under meadow vegetation. Since microorganisms are sensitive to changes in physic-chemical environmental conditions and, at the same time, participate in key processes of nutrient cycling and transformation in soils, the study of shifts in the structure of soil microbiocenosis can be a step in understanding and assessing the direction of soil-biological processes in soils, ensuring, in turn, the functioning and sustainability of the soil ecosystem. Thus, the identification of the main drivers of microbiome composition, as well as microbial taxa marking the change in soil environment occurring because of man-made forest plantations is an interesting research problem. For this purpose, the scope of this study was to analyze shifts in the taxonomic structure of prokaryotic communities of mountain-meadow soils under the influence artificial planting of larch (*Larix sibirica* Ledeb.), in comparison with control soil under the cover of grassland meadow vegetation. The effect of larch planting on both pH and organic matter content, which is reflected in the number and abundance of soil microorganisms, seems to cause significant changes in the composition and structure of microbiomes. So, the objectives of the study included: 1) identification of microbial taxa associated with two biotopes–the soil under larch and under zonal meadow vegetation; 2) analysis of the influence strength of factors–vegetation type, pH, Corg, depth of sampling, on the structure and diversity of the prokaryotic community of mountain-meadow soils.

## Materials and methods

### Description of the site of soil sample selection

The Ai-Petri plateau belongs to the system of western yailas of the Mountainous Crimea with absolute heights of 1,100–1,300 m above sea level. The climate of the plateau according to the Ai-Petri weather station (1,180 m) is characterized by an average annual precipitation of 1,052 mm, an average annual temperature of 5.7°C, an average temperature of -3.8°C in February, and 15.5°C in July. Most of the precipitation (62%) falls during the cold season from November to March [7].

Yailas are hilly upland plateaus with numerous karst sinkholes, wells, mines, and caves [8].

The soil cover of the plateau under man-made forests is represented by mountain meadow soils (Leptic Phaeozems according to WRB [9]) on leached weathering products of the Upper Jurassic limestones. Until the second half of the 20th century, the territory of the plateau with the most fertile soils was actively used for pastures and vegetable gardens, and until now as haylands, which led to a noticeable degradation of the soil cover, primarily to the loss of a significant part of organic carbon.

Artificial larch plantations on the Ai-Petri were created in 1963–64 and by the time of the study they had reached the age of 54–55 years [6]. The trees were planted in accordance with plant-close system of 3 × 0.5–1 m, which subsequently led to numerous plant mortalities and suppression of the remaining plants. By now, only the upper thirds of the crowns have remained green. In addition to larch, the forests contained hamate pine (*Pinus kochiana* Klotzsch ex K. Koch), linden (*Tilia cordata* Mill.), and ash (*Fraxinus excelsior* L.). Many mountain ashes (*Sorbus domestica* L.) were found in the undergrowth. The crown density in general was 0.7–0.8, the projective cover of the ground cover was about 50% and consisted of the slender false brome (*Brachypodium sylvaticum* (Huds.) P. Beauv), cocksfoot (*Dactylis glomerata* L.), mountain willow herb (*Epilobium montanum* L.), herb Robert (*Geranium robertianum* L.), nipplewort (*Lapsana intermedia* M. Bieb.), bluegrass (*Poa nemoralis* L.), big-sting nettle (*Urtica dioica* L.), and self-seeding of tree species. The litter thickness did not exceed 2–3 cm.

In terms of the composition of vegetation, the areas of the plateau adjacent to the forest plantations belong to the meadow steppe, typical for the Ai-Petri yaila. In accordance with our data, the dominant species for meadow (grassland) communities are gramineous plants: helictotrichon (*Helictotrichon schellianum* (Hack.) Kitag.), dithering-grass (*Briza elatior* Sibth. & Sm.), couch-grass (*Elytrigia repens* (L.) Gould), bluegrass (*Poa pratensis* L.), cocksfoot, purple-stem (*Phleum phleoides* (L.) H. Karst.), bluegrass (*Elytrigia strigosa* (M. Bieb.) Nevski), as well as milfoil (*Achillea setacea* Waldst. & Kit.), common betony (*Betonica officinalis* L.), common St. John’s wort (*Hypericum perforatum* L.), dog-mint (*Clinopodium vulgare* L.), wild strawberry (*Fragaria viridis* (Duchesne) Weston), whip-tongue (*Galium mollugo* L.), lungwort (*Pulmonaria obscura* Dumort.), and some other species [6].

### Soil sampling and analysis of physic-chemical parameters

**Fig 1:**
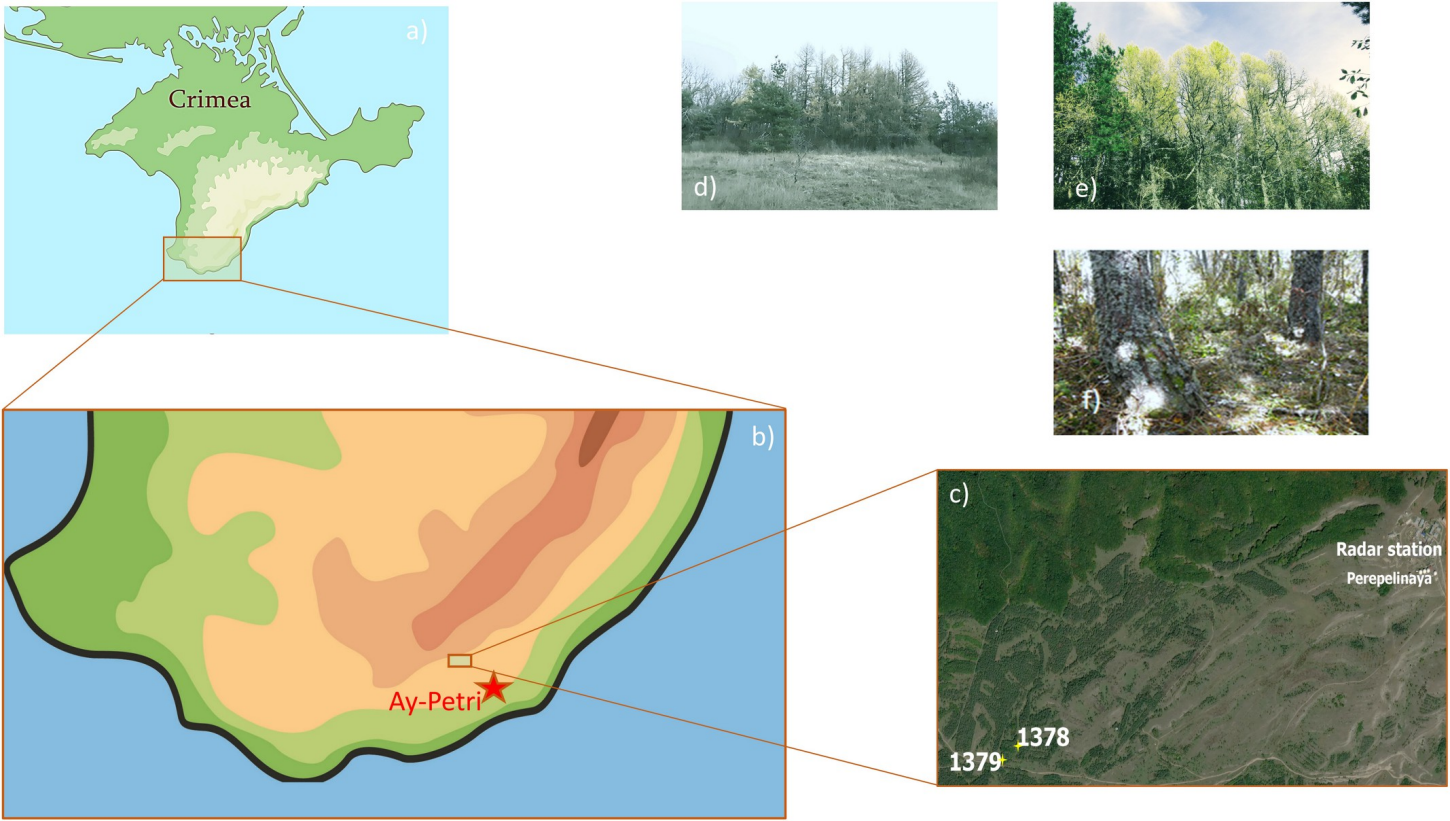
Soil sampling map and photos of the objects. Soil sampling point–a-c); photos of the sampling sites: meadow (d), larch (e), and canopy under a larch forest (f).

Soil pits were made under larch plantations (pit № 1378, 44.475492° N 33.996967° E) and in the adjacent meadow (pit № 1379, 44.475315° N 33.995926° E) to a depth of 25 cm, samples from which were taken with a continuous column every 5 cm (Fig 1).

The granulometric composition was determined by the pipette method after pyrophosphate dispergation of soils, with further identification of the texture class [10]. pH values were measured in aqueous suspension at 1:2.5 ratio (by the pH-metric method). Hydrolytic acidity was determined in by alkaline titration method. The content of exchangeable bases was detected by extraction with 0.2 N NH4Cl solution. Total organic carbon (Corg) was evaluated by Tyurin indirect oxidation method [11]. The content of mobile forms of Pb, Mn, Cu, and Zn was determined in 1M ammonium acetate extract with pH 4.8 [12].

### Total DNA isolation and NGS-sequencing

Soil samples were stored at -80°C before DNA extraction for one year. Isolation of total DNA was carried out from 0.5 g of soil using a commercial FastDNA® SPIN kit for soil (MP Biomedicals, Germany) according to the manufacturer’s protocol. Soil samples were added to test tubes containing lysing mixture, which also contained a silica matrix and glass beads of various diameters. The samples underwent physical destruction in a FastPrep® apparatus and treated with a protein precipitating solution (150 μl of 3 M CH3COOK and 4% of glacial acetic acid). DNA was bound by a DNA binding matrix (1 ml of “glass milk” diluted 1:5 with 6 M guanidine isothiocyanate), washed with SEWS solution (ultrapure 100% ethanol and 0.1 M sodium acetate), and finally eluted in water free of DES. The purified DNA extracts were stored at -20°C.

The libraries preparation included amplification of the target fragment of the V4 region of 16S rRNA gene using universal primers (515F - GTGCCAGCMGCCGCGGTAA / 806R - GGACTACVSGGGTATCTAAT) [13] together with linkers and unique barcodes. PCR was carried out in a T100 Thermal Cycler (BIO-RAD Laboratories, USA), in 15 μL of a reaction mixture containing 0.5 units of Q5® High-Fidelity DNA Polymerase (New England BioLabs, USA), 1X Q5 Reaction Buffer, 5 pM of each primer, 3.5 mM of dNTP (Evrogen, Russia) and 1–10 ng of DNA matrix. The PCR protocol included denaturation at 94°C—1’, amplification of the product during 35 cycles (94°C—30", 50°C—30", 72°C—30") and final elongation at 72°C—3’. Further sample preparation and sequencing were carried out by using Illumina protocol (“16S Metagenomic Sequencing Library Preparation”) on Illumina MiSeq (Illumina Inc., USA) using a MiSeq Reagent Kit v3 (600 cycle) with two-sided reads (2 * 300 n) (Illumina Inc., USA).

### NGS-data processing

Data processing included the removal ofservice sequences by using cutadapt program [14], as well as denoising, combining paired reads, and removing of chimeras using the dada2 package [15] implemented in the R software, The processing was performed in accordance with authors’ recommendations [15]. Service sequences (barcodes) were cut by Illumina, primers were deleted during trimming of sequences. Sequences that were assigned to mitochondria or chloroplasts were removed as well. For trimming F515/R806 primers, the following parameters were set manually: `truncLen = c(220,180), trimLeft = c(19, 20)`; the standard quality filtering parameters were used (maxN = 0, truncQ = 2, rm.phix = TRUE and maxEE = c(2, 2)). Common source of samples allows us to use pooling and set `pool = TRUE`. Sequences were rarefied to minimal depth in 13623 reads per sample. The main analysis of the results was carried out using the phyloseq (v1.30.0) packages [16] in R (v3.6.3).

Taxonomic classification of the obtained ASV (amplicon sequence variant) sequences was also carried out using 132 releases of the SILVA database containing data for the SSU (Small SubUnit) rRNA gene [17]. Further processing, including the build-up of a phylogenetic tree using the SEPP algorithm [18], the calculation of alpha- and beta-diversity were carried out within the QIIME2 package [19] and the plugins implemented in it. To assess alpha diversity, the diversity indices reflecting the observed ASVs and predicted species richness (Chao1), Faith’s PD (Phylogenetic Distance), the degree of evenness (Shannon), and dominance (Simpson) were calculated. Beta-diversity analysis was presented by scaling of the Bray-Curtis pair-wise distance matrix using the NMDS (Non-Metric Multidimensional Scaling) method.

### Statistical analysis

The results were statistically processed using the R software package (https://cran.r-project.org/). The significance of differences in taxa richness and diversity indices was assessed using the Student’s t-test. Correlation analysis was performed based on the Pearson correlation coefficient. p-values refer to F-statistics of the significance test of the weighted linear trend: we used p < 0.05 to determine significant difference.

Microbial communities in addition to beta-diversity were tested for significant difference between Larch and Grassland groups using the following statistical tests–non-parametric multivariate analysis of variance (NPMANOVA) and analysis of similarities (ANOSIM) with Bray-Curtis dissimilarities measure and 9999 permutations.

To examine the variation of biodiversity with depth we used generalized biodiversity index which combines traditional indicators of biodiversity and reflects ecosystem multi-functionality—Shannon index, Simpson index, Phylogenetic Diversity, and total amount of observed ASVs. Generalized biodiversity index is obtained by standardization of the scores of biodiversity indices (z-score normalization) and is frequently used in ecological and soil investigations [20, 21]. It provides convenient and clear illustration of the dynamics of overall diversity along with soil depth in both soils–under larch and grassland.

To identify taxa, the richness of which changed significantly due to phytocenotic conversion, as well as to study and visualize the effect of physic-chemical properties of the studied soils on the proportion of dominant taxa, the principal component analysis was used (using the prcomp function). The analysis was carried out for 59 “dominant” ASVs, the proportion of which exceeded 1% in at least one sample.

## Results

### Characteristics of the physic-chemical parameters of the studied soils

According to particle-size distribution analysis, the soil under larch belongs to light clay, and under grassland vegetation—to heavy loam (S1 Table). No regularities in the pro-file distribution of the silt fraction were noted, which is due to the plowing of the soil before tree planting. The mixing of the upper 20-cm layer is also evidenced by the relatively uniform distribution of C org, especially in the soil under larch, where the rate of humus accumulation in the 0–10 cm layer was lower than under herbaceous (grassland) vegetation.

The growth of larch contributed to a stronger acidification of the soil in comparison with grassland vegetation. By the time of the research, the pH and the sum of exchangeable bases in the soil under the forest were lower than in the grassland, and HA was almost twice as high. An increase in acidity stimulated the mobilization of several elements from the mineral part of the soil, the concentration of mobile forms of which in the soil under larch was 2–3 times higher than under grassland.

### Biodiversity analysis

**Fig 2:**
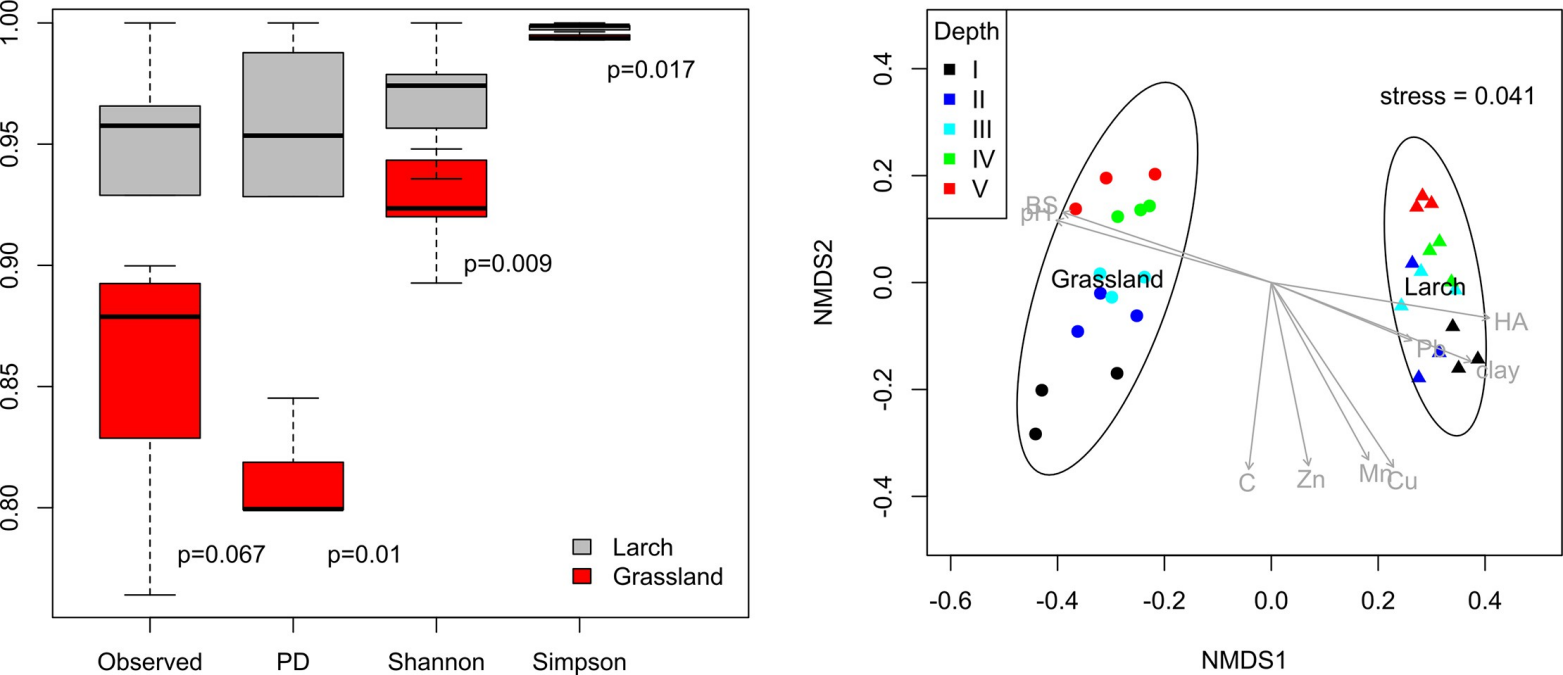
**a. Diversity indices of mountain-meadow soil under grassland vegetation (Grassland) and under artificial plantations of larch (Larch).** Index values* were normalized to their corresponding maximum values. *Raw values of the alpha diversity indices are given in S2 Table. **b. Beta-diversity (based on Bray-Curtis distance matrix) of soil microbiomes under grassland vegetation (Grassland) and under artificial plantations of larch (Larch).** Roman numerals correspond to the depth of sampling: I—0–5 cm, II—5–10 cm, III—10–15 cm, IV—15–20 cm, V—20–25 cm. For stress plot of MNDS-analysis please refer to S3 Fig.

Analysis of alpha-diversity indices demonstrates a significant difference in the structure of prokaryotic communities in the studied biotopes–an increase in all diversity indices in the soil under larch (Fig 2A). The effect of sampling depth was also determined for the soils of both biotopes (S2 Fig): a statistically significant increase in biodiversity at depths of 10–20 cm was noted. For the soil under larch, a statistically significant maximum of the phylotypes (ASVs) number and their phylogenetic diversity was recorded in the upper layer of 0–5 cm, and a minimum in the 20–25 cm layer of (S2 Fig and S2 Table).

When analyzing beta-diversity (Fig 2B), the influence of the vegetation type was also clearly noticed, while the type of dominant vegetation (grassland vegetation and larch forest), had a more significant impact on the structure of the prokaryotic community than the sampling depth. Microbial communities under Larch and Grassland show statistically significant differences using both NPMANOVA (p = 0.0085) and ANOSIM tests (ANOSIM statistic R is 1 with p = 0.0064).

### Changes in the taxonomic structure of microbiomes resulting from man-made forests of larch

**Fig 3:**
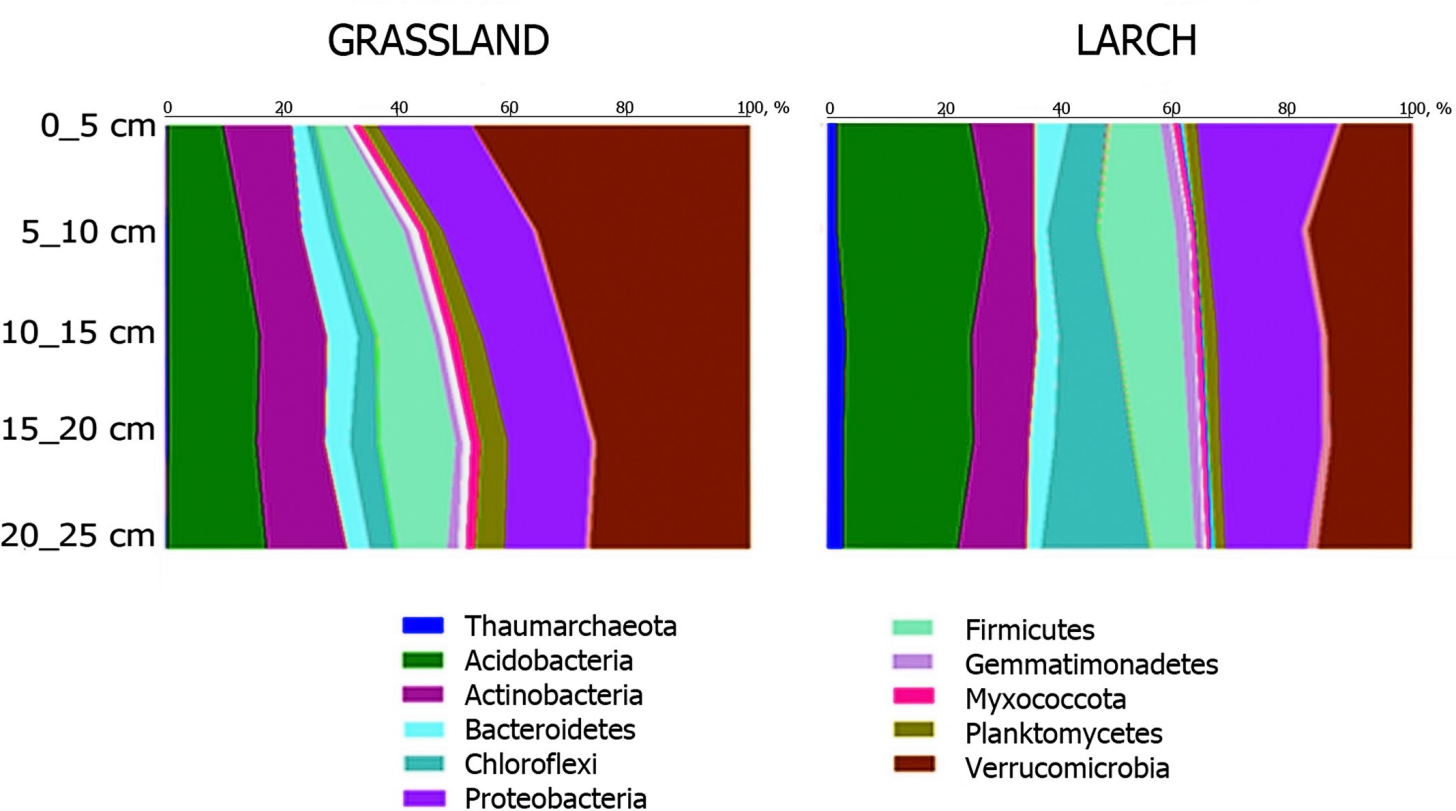
Taxonomic structure of soil microbiomes at the level of prokaryotic phyla*. *Only phyla with a share of more than 1%** in the community are represented. **The proportional values of dominating phyla in each soil sample are indicated in S3 Table.

Prokaryotes from 10 bacterial and 1 archaeal phylum dominated in the studied microbiomes: bacteria: *Verrucomicrobia* (24.1%), *Acidobacteria* (18.5%), *Proteobacteria* (16.4%), *Actinobacteria* (10.9%), *Firmicutes* (10.2%), *Chloroflexi* (7.0%), *Bacteroidetes* (3.6%), *Planctomycetes* (2.6%), *Gemmatimonadetes* (1.6%), *Myxococcota* (1.0); archaea: *Thaumarchaeota* (1.1%) (Fig 3).

In the microbiomes of soil samples under larch, a tendency of an increase in the relative proportion of archaea of the phylum *Thaumarchaeota* (in 6 times) and bacteria of the phyla *Chloroflexi* (in 4.5 times) and *Proteobacteria* (in 1.3 times) was determined. On the contrary, the proportion of bacteria of the phyla *Planctomycetes* (1.8 times), *Verrucomicrobia*, and *Actinobacteria* (1.2 times) significantly decreased here. A tendency to a decrease in the richness and an increase in the number (1.7 times) and diversity of *Acidobacteria* was noted.

In both biotopes, a decrease with depth in the proportion of proteobacteria (mainly of *Alpha-* and *Gammaproteobacteria* groups) and an increase in the proportion of bacteria of the phylum *Chloroflexi* were observed (Fig 3).

**Fig 4:**
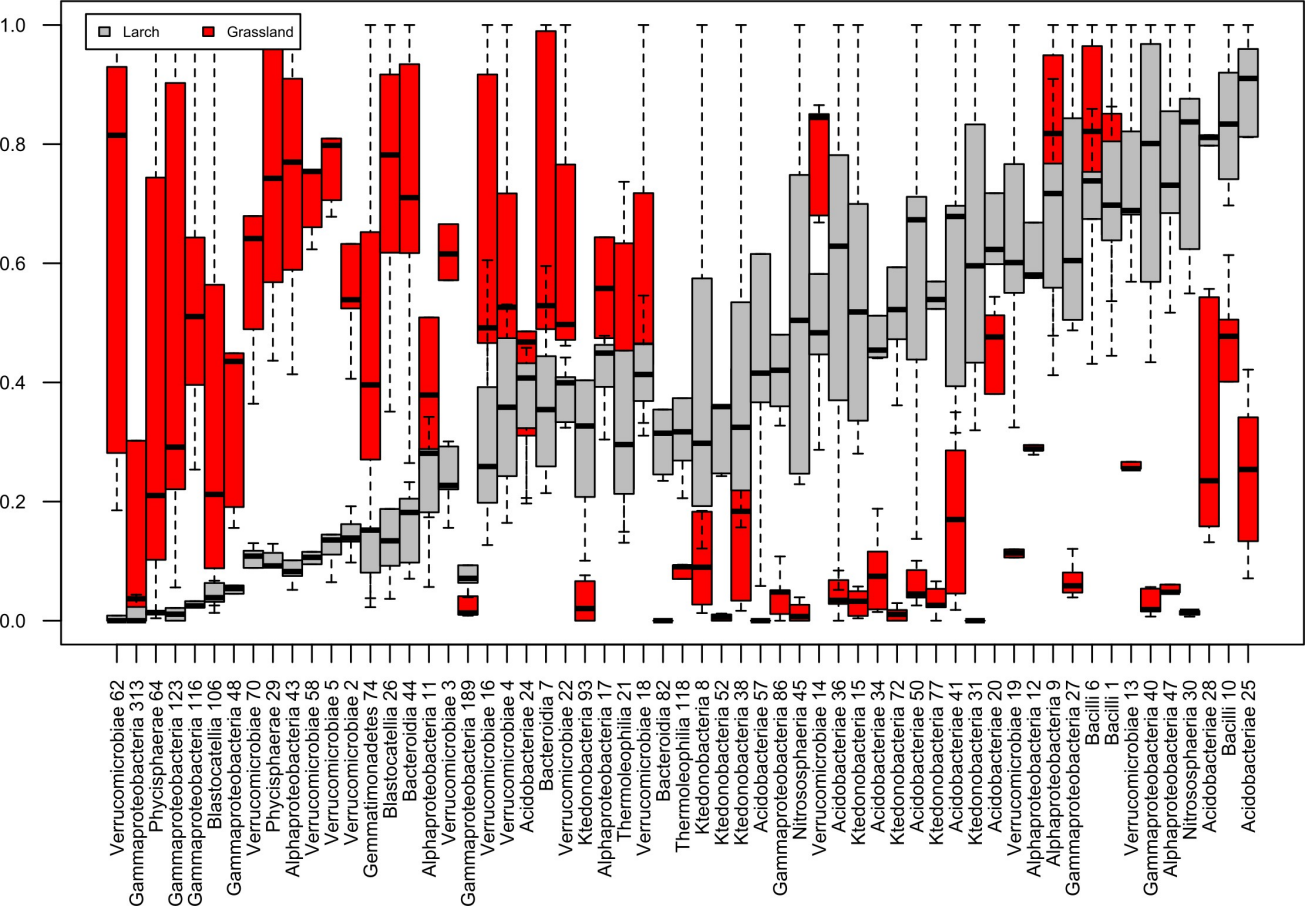
Abundance of dominant phylotypes in the soil microbiome under grassland vegetation (Grassland) and artificial plantations of larch (Larch). Taxonomic identification of phylotypes* is given at a high taxonomic level (at the level of prokaryote classes). Numbers denote ASV numbers in the original dataset. The boxplot is an average of 15 samples for each taxon, (i.e., without considering the depth factor). The values of the proportion of each phylotype are normalized to the maximum from both biotopes to allow comparison of the content of individual taxa between biotopes. S4 Table shows the numerical values of the averages (dash in the boxplot) to Fig 4, sorted in descending order of difference for the taxa between biotopes. *The complete taxonomic assignment of 59 ASVs is presented in S4 Table.

To identify the taxa marking phytocenotic conversion, as well as those which abundance is related to depth and, at the same time, to changes in physicochemical parameters, 59 dominant phylotypes were selected from the entire dataset. The threshold for identifying phylotypes was 1% of their proportion in at least one of the analyzed biotopes (Larch or Grassland) (Fig 4).

In the soil under larch, an increase in the proportion of phylotypes of bacteria from the orders *Ktetonobacterales (Chloroflexi)* and *Bacillales (Firmicultes)*, as well as archaea from the family *Nitrososphaeraceae (Thaumarchaeota)* was noted. Moreover, plant community conversion led to the shifts in the composition and ratio of acidobacterial groups. When the dominant part of the soil community under grassland vegetation includes representatives of the *Blastocatella* class (Fig 4), the elimination of this group of prokaryotes was observed in the soil under larch. The presence of acidobacteria Subgroup_2, as well as order *Acidobacteriales* and *Bryobacterales* (Fig 4 and S4 Table) characterized the dominant community of the Larch biotope.

In the soil under grassland vegetation, a significantly larger proportion of phylotypes of orders *Chthoniobacterales (Verrucomicrobia)*, *Rhizobiales (Alphaproteobacteria)*, *Burkholderiales (Gammaproteobacteria)*, as well as family *Phycsphaeraceae (Planltomycetes)* (Fig 4 and S4 Table) was determined.

The observed statistically significant division in the content of dominant phylotypes even when mixing samples from different depths (i. e., without considering the depth factor) suggests that the plant community is a stronger factor determining the difference in the structure of microbiomes, in comparison to depth. Nevertheless, the depth factor can cause a more subtle effect on the structure of the microbial community within each biotope (for example, the size of the “box” may indicate which taxa have the greatest variation in richness with depth (Fig 4)).

### Analysis of the relationship between the taxonomic structure of prokaryotic communities and the physic-chemical conditions of the soils of the studied biotopes: Taxonomic markers of phytocenotic conversion

Biotopes generally differ in the chemical composition of soils; the ranges of values for all indicators, except for carbon, generally did not overlap (S1 Fig). Under grassland vegetation, soil samples were characterized by relatively low values of all investigated parameters, except for the number of bases (BS) and pH, where, on the contrary, lower values of these indicators were observed in the Larch biotope.

**Fig 5:**
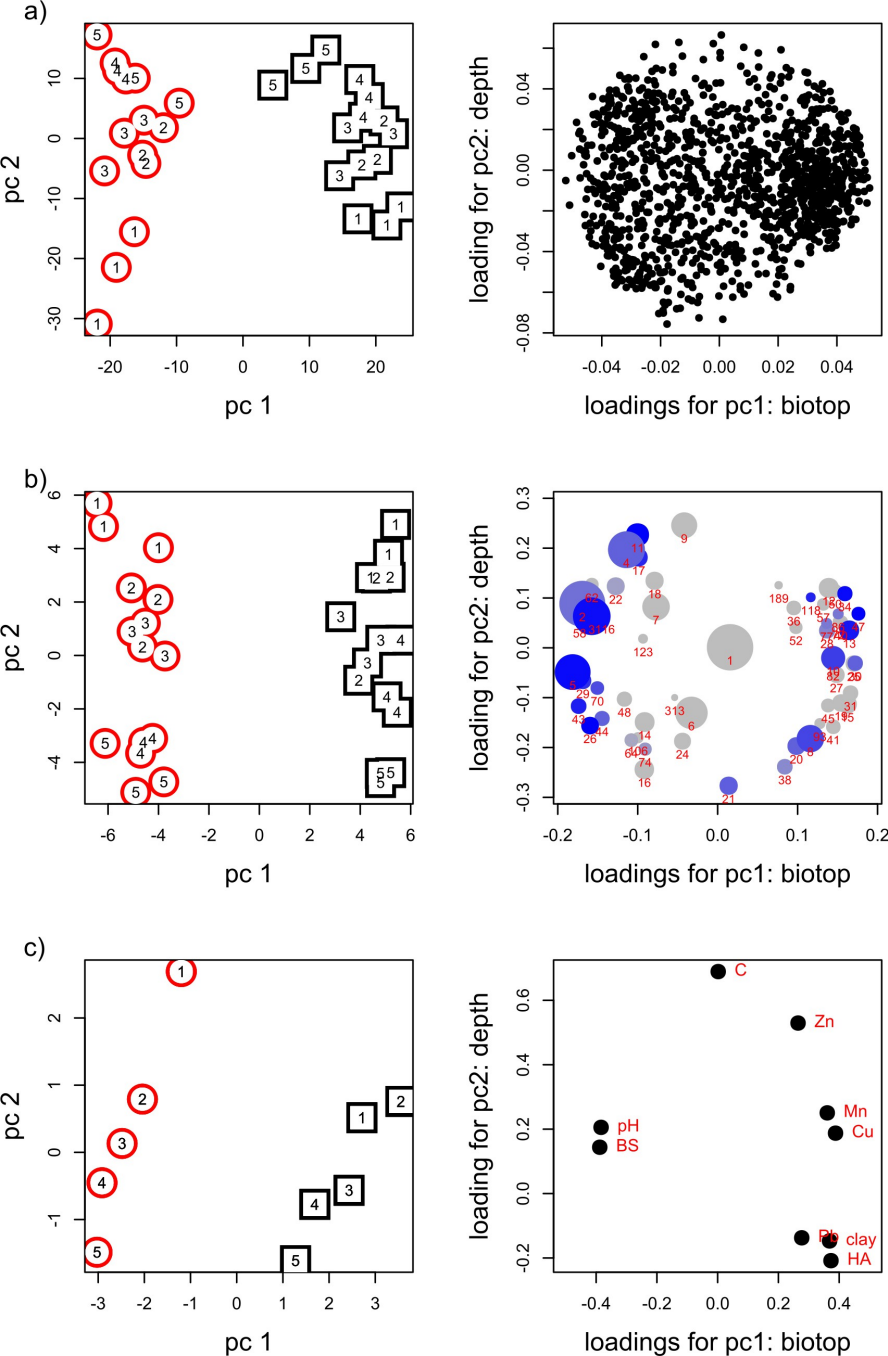
PCA analysis of the taxa abundance and values of physic-chemical factors. PCs for all taxa (a), dominant taxa (b) and chemical factors (c) are presented. Left column shows sites—grassland vegetation (red circles) and larch plantations (black squares); numbers in the left panel indicate the depths of sampling: 1- layer 0–5 cm, 2–5–10 cm, 3–10–15 cm, 4–15–20 cm, 5–20–25 cm. On the right column PCA loadings are presented: in panel (b) the area of the circle is proportional to the fraction of ASV, the intensity in blue shows the quality of the trend (R2) for the taxa that were found to have statistically significant trends with the chemical factors. Please find the Loading values for PCs of both chemical factors and microbial taxa in S4 Fig.

Principal component analysis was carried out for all taxa, dominant taxa and for soil physic-chemical parameters. A clear difference in biotopes across the PC1 axis was determined. The positive PC1 values correspond to larch samples, and negative ones–to the soil under grassland vegetation. PC2 for both taxa and chemical parameters has statistically significant trends with depth for both biotopes (Fig 5 and S4 Fig). Samples of 0–5 cm and 5–10 cm layers correspond mainly to positive values of the PC2 axis, while deeper layers (10–25 cm) are located mainly in negative values area of the PC2 axis. For both studied biotopes, a statistically significant linear trend in the dependence of PC2 of both taxa (p = 0.0001) and chemical parameters (p = 0.0004) on depth was found (S4A Fig).

The analysis revealed the principal contribution of the dominant set of taxa both to the difference between biotopes and to changes caused by sampling depth (PC1 and PC2, respectively, Fig 5B) Loading values for dominant taxa (Fig 5B, right) show up to be maximal among all taxa (forming the outer circle of the cloud of all taxa on Fig 5A, right).

In PC1 of physic-chemical properties, which determines the differences in biotopes, the hydrolytic acidity, the content of mobile forms of trace elements and sludge clay make a positive contribution, while pH and amounts of bases make a negative one. In PC2, which determines the differences in the values of physic-chemical parameters with depth, the maximum contribution is made by the content of organic carbon and zinc (Zn) (S4B Fig).

**Fig 6:**
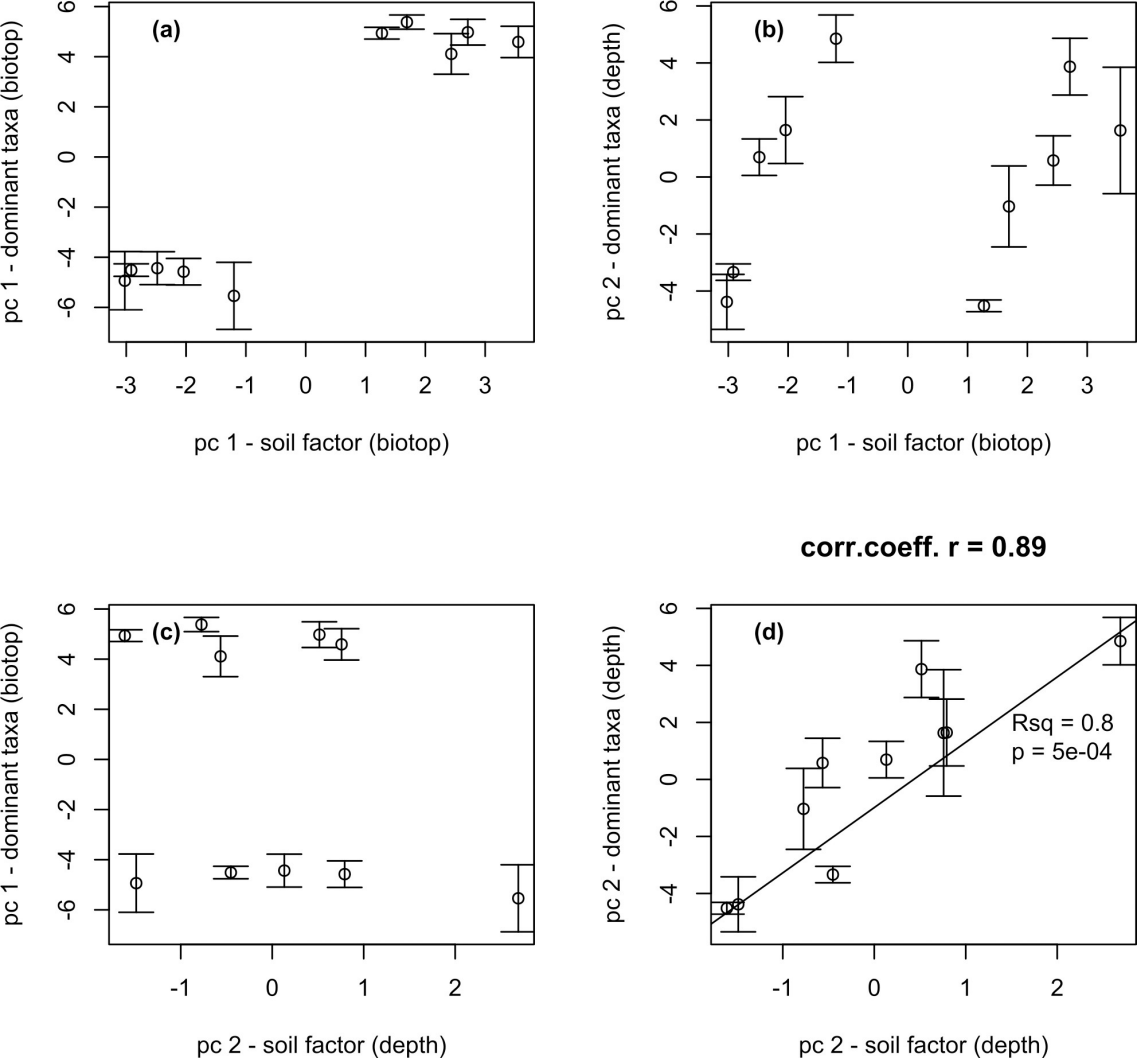
Correlation analysis between the two major components of taxa and the two major components of chemical indicators.

To identify the relationships between the structure of the community as a whole and soil physic-chemical, an analysis of the correlations between the two Principal Components of taxa and the two Principal Components of physic-chemical parameters was carried out. Fig 6 shows a significant correlation between taxa and physic-chemical parameters both at the level of differences between biotopes (Fig 6A) and within each biotope (Fig 6D). Strong correlations were found between PCA components for taxonomic and chemical composition, these components also correlate strongly with biotopes (both PC1, while not with its orthogonal PC2) and sampling depth (both PC2 while not with its orthogonal PC1). This means that biotopes and depth both vary in taxa as well as in chemistry, i.e. depth dominant taxa variation correlates with soil chemistry distribution with depth (Fig 6D). The correlation coefficient was 0.94 and 0.89, respectively.

**Fig 7:**
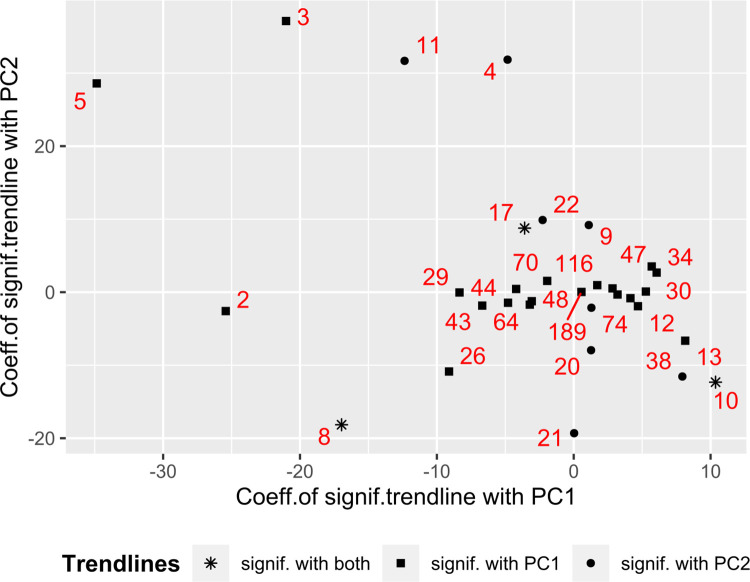
The summarized graph for the values of the coefficients of significant linear trends. Only taxa that have a significant trend with at least one of the components of soil properties are shown. The greater the slope coefficient of the trend, the stronger the dependence of taxon abundance on the component.

In PC1 of taxa (Fig 7 and S4 Fig) a negative contribution is made by the richness of phylotypes *Verrucomicrobia*, acidobacteria of the *Blastocatella* class, family *Phycisphaerae (Planctomycetes*) and order *Burkholderiales (Gammaproteobacteria)*, and a positive one–by the richness of archaea of family *Nitrososphaeraceae*. In PC2, which determine the differences in the richness of taxa with depth, the phylotypes of *Verrucomicrobia* and order *Rhizobiales* make a positive contribution, and the richness of family *Ktedonobacteraceae (Chloroflexi)* and *Bacillales (Firmicutes)* make a negative one.

For some taxa, individual significant correlations with physic-chemical parameters (in the form of their PC1 (distinguishing biotopes) and PC2 (distinguishing depths of sampling)) were revealed (S4 Fig).

## Discussion

The identification of the main factors and parameters of the environment (that determine the structure and functioning of the soil microbial community) is a key point that does not lose its relevance in the microbial ecology. Of all the factors affecting the microbiome, the structure and composition of the plant community has a significant and yet complex influence. Changes in the composition and diversity of the plant community have a direct and indirect influence on the structure of the soil microbiome. In some cases, the structure of the microbial community was mainly determined by the species composition of the vegetation [22, 23]. The direct effect of plants is determined by the release of organic matter and the composition of root exudates, which attract microorganisms and thus form an associative symbiosis. The indirect effect arises due to changes in physic-chemical parameters: aggregate composition, pH of the upper soil layer, organic carbon content, etc. [4]. Thus, human-directed changes in the composition of the plant community, such as the removal of forest or the creation of artificial forest strips, may certainly affect the structure and functioning of the soil microbial complex. In this context, the relevant research objective is to analyze which factors have the strongest influence on the abundance of individual taxa and whether the specificity of prokaryotic patterns is more related to the type of dominant vegetation composition, to the depth of sampling or to both factors.

For the period from 1957 to 1970, forest plantations on the Ai-Petri plateau were created on mountain-grassland soils, which had previously been actively used as hayfields and vegetable gardens. Of all the planted species, larch had the most noticeable effect on the physic-chemical and chemical properties of the soil, which is due to the strong acidifying effect of this species [4, 5]. In this regard, larch plantations were selected as an object of research.

The type of plant community had a significant impact on the structure of the microbiome. An increase in diversity was observed under larch, and the microbiomes inhabiting soil under forest plantations were distinguished by the highest evenness (even at the level of dominant taxa), in comparison with soil microbiomes under grassland vegetation, where clear dominants were found (Figs 3–5). A certain stratification of both biodiversity and the taxonomic structure of microbiomes in soils under larch with depth was observed, which indicates a more significant effect of forest plantations on the microbiome, since the upper 20 cm soil layer was mixed before planting larch (see above).

pH of the soil solution is known as the main factor affecting the composition and diversity of the soil microbiome [24–28]. Most of the studies demonstrate a positive correlation between microbial diversity and pH [22, 25–27], while the maximum diversity was often observed in soils with neutral pH values and lower values, respectively, in soils with pH in a more acidic area [29–31]. Our results indicate a tendency towards an increase in diversity in the upper soil layers of larch (S2 Table and Fig 1), also characterized by lower pH values of the soil solution compared to the mountain grassland soil under the cover of grassland vegetation (Table 1). It should also be noted that most of the research show the results of soil analysis in a wide range of pH values: 4–8 [29, 30], 5–8 [26], while in our study pH changed, albeit significantly (under the conditions of forest plantations, a decrease was noted on average from 5.26 to 4.81), but the range was relatively narrower. At the same time, in the soil samples under larch, a significant heaving in the granulometric composition was observed, which, in turn, can reduce the determining influence of pH on the rate of microbial diversity. Some data also indicate to the sorption of DNA on clay particles [32], which may indirectly contribute to the overestimation of the results on the estimates of the biodiversity of soils with a high content of fine particles. On the other hand, man-made larch forests were formed no more than 50 years ago, which suggests a transitional nature of the larch forest ecosystem, compared to the climax system of mountain grassland soil formed for a long time under the cover of zonal (grassland) vegetation. According to the ideas of Clements [33], one of the main properties of the climax system is stability (including community composition), suggesting that there are clear dominants in the community, which are as well least susceptible to changes in the physical environment, in contrast to communities with lower stability. Such a high stability of “climax” communities leads to a relatively low species diversity since the time between radical changes in the ecosystem is long enough to allow one or more species to dominate. Our work revealed clear dominants in the structure of prokaryotic communities in soil under meadow vegetation, while the proportions of microbial taxa in soil under larch differed not so dramatically, which is probably due to dynamic changes in the architecture and organization of ecological niches occupied by microorganisms and, as a result, colonization of new habitats by diverse microorganisms. The latter may possibly lead to an increase in biodiversity in soil under larch. At the same time, these are only indirect signs of the "transitional nature" of the community, and clear conclusions require the analysis of chronosequences, allowing the analysis of the dynamics of the composition and biodiversity of the soil microbiomes [34].

**Table 1:** Soil properties under artificial larch plantations and grassland vegetation. The values are presented as Mean ± SD.

Soil pit	Depth, cm	Silt, %	рН	Corg, %	HA	Base sum (BS)	Pb	Mn	Cu	Zn
					cmol(+)*kg-1		avialable, mg*kg-1			
1378 Larch 44.475492° N 33.996967° E	0–5	44±1	4.79±0.02	3.38±0.18	14.3±0.2	15.8±0.7	2.09±0.06	21.3±5.4	0.16±0.02	1.17±0.11
	5–10	43±1	4.74±0.01	3.18±0.06	13.1±0.1	13.8±0.2	1.95±0.41	23.2±3.3	0.18±0.02	1.03±0.06
	10–15	39±4	4.94±0.03	3.41±0.11	14.0±0.3	14.5±0.4	5.13±0.78	19.0±4.4	0.15±0.02	0.75±0.13
	15–20	40±2	4.75±0.02	3.27±0.10	14.5±0.2	13.9±0.3	1.74±0.11	16.1±4.1	0.14±0.01	0.69±0.15
	20–25	41±5	4.80±0.01	2.99±0.02	15.0±0.2	14.8±0.7	2.09±0.23	12.7±2.1	0.12±0.01	0.52±0.08
	Mean	41	4.80	3.25	14.2	14.2	2.60	18.5	0.15	0.83
1379 Grassland 44.475315° N 33.995926° E	0–5	26±2	5.22±0.03	4.15±0.15	8.7±0.1	22.5±4.4	1.00±0.24	12.9±2.0	0.10±0.02	1.18±0.10
	5–10	27±1	5.17±0.02	3.65±0.03	8.4±0.2	22.3±3.3	0.76±0.23	9.8±1.1	0.10±0.02	0.50±0.04
	10–15	32±3	5.30±0.02	3.38±0.15	7.2±0.1	22.0±2.8	0.69±0.17	8.7±1.4	0.07±0.02	0.33±0.04
	15–20	26±2	5.29±0.01	3.16±0.10	8.0±0.1	22.3±3.3	0.66±0.16	7.9±1.4	0.06±0.02	0.28±0.06
	20–25	28±3	5.35±0.02	2.87±0.22	7.7±0.2	23.0±3.5	0.63±0.15	7.4±1.3	0.05±0.01	0.25±0.04
	Mean	28	5.26	3.44	8.0	22.4	0.79	9.3	0.08	0.51

The change in the type of plant community significantly affected the richness of acidobacteria. Thus, a shift in pH to the “acidic” values leads to an increase in the proportion of both all acidobacteria and their individual representatives: an increase in the proportion of bacteria of the Subgroup_2 and order *Bryobacterales*, as well as the reduction of bacteria of the *Blastocatella* group. Representatives of the latter group are typical inhabitants of arid soils of warm latitudes [35]; their richness also often positively correlated with the reaction of the soil solution in the region of neutral or alkaline pH [36, 37] characteristic for samples of native mountain grassland soil (Table 1). Representatives of the Subgroup_2, on the contrary, were associated with more acidic soils [38], which may explain the increase in their richness in the soil under larch. Thus, in microbiomes, we can observe a clear trend towards acidification of the microbiome and its approximation to podzolic soils. It is also worth noting that the studied soils demonstrate some tendency of “podzolization” by shifts in physic-chemical properties, expressed, in addition to pH changes, in the presence of silica powdering in the upper layer, as well as a tendency towards eluvial-illuvial differentiation of the soil layer by the amount of organic matter and bases (Table 1). The diversity pattern observed in the soil under larch show a sharp decrease in biodiversity in the 5–10 cm layer, which may also indirectly indicate the beginning of the podzol-forming process: when studying the chronosequences of podzolic soils, the manifestation of this process was accompanied by a significant decrease in taxonomic and phylogenetic diversity in the eluvial layer [38].

In the soil under man-made larch stands, a significant decrease in the richness of *Verrucomicrobia* was determined. This group of bacteria is, according to literature, sensitive to the concentration of organic matter in soils [39, 40], which explains its appearance in the upper root-inhabited soil layers. This group was found as well in chernozems under the cover of zonal vegetation [40], which, in turn, indirectly indicates that the optimum of this group is around neutral pH values. Our results demonstrate a positive correlation between the richness of verrucomicrobial phylotypes both with the amount of organic matter and with an increase in the pH of the soil solution (Fig 4, and S4A and S4B Fig), which, in general, also explains the large proportion of verrucomicrobia in the soil under grassland vegetation (S3 Table).

The influence of the depth of sampling was manifested as a subordinate factor within the framework of separately taken soil microbiomes under grassland vegetation and larch plantations. The rhizosphere bacteria of the order *Rhizobiales* and *Burkholderiales* demonstrated a predominant correlation with the upper layers of the native mountain grassland soil, rich with organic matter. This result is generally consistent with the ecological characteristics of this group [41].

The richness of *Chloroflexi* phylotypes of the *Ktetonobacteraceae* family correlated with depth. Most bacteria of this group are characterized by an oligotrophic type of nutrition and are facultative or obligate anaerobes, which contributes to the maintenance of their viability in deep mineral layers depleted in available forms of nutrients. In our studies, bacteria of the family *Ktedonobacteraceae* were mostly associated with the larch biotope (Fig 3 and S4 Fig), characterized by an increase in finely dispersed fractions and negatively correlated with the amount of available organic matter (S4 Fig). The latter may explain their relative increase in the lower soil layers of this habitat (Fig 4). To a certain extent, the phylotypes of *Firmicutes* also negatively correlated with organic matter. This group of bacteria is characterized by the ability to form spores, suggesting their surviving in unfavorable conditions [42]: in relatively low values of soil organic matter reserves and acidification of soil solution, which are characteristics of soils of a larch forest.

Changes in the soil ecosystem under the influence of artificial plantations of larch are reflected in shifts in the taxonomic structure of communities. The latter is due to a complex effect of the plant conversion on the whole spectrum of physic-chemical soil parameters, particularly due to the decrease in organic matter, as well as significant acidifying effect of larch litter decomposition and accompanying processes of mobilization of trace elements (Table 1). Representatives of *Proteobacteria*, *Firmicutes*, and *Chloroflexi* phyla are known to be resistant to an increase in the proportion of trace elements, also referred to the heavy metal category [43]. This can partly explain the relative increase in the abundance of these microorganisms in soil samples under larch. Additionally, some data exist supporting the resistance of archaea in soils contaminated with heavy metals, which can also theoretically explain the increase in their abundance in larch biotope [44].

Meanwhile, in general, considering the dynamical behavior of the soil ecosystem and the interrelation of physicochemical parameters with each other, identification of the relationship between the action of a particular factor (group of factors) and the abundance of certain taxa becomes challenging, especially if we take into account the composite nature of the NGS data [45]. The conclusions obtained may thus have a somewhat speculative appearance and need further verification by carefully considered model experiments, including the use of differential approaches. In particular, the use of taxon-specific primers would allow a more complete characterization of the shifts in the archaea structure, the proportion of which in the analyzed samples was presumably underestimated.

Nevertheless, the identified patterns in the structure and diversity of soil microbiomes demonstrate the key ecological role of plants as an ecological driver of soil microbiocenosis, expressed in a significant impact of artificial forest plantations on the prokaryotic communities of mountain-meadow soils. The latter is reflected in the stratification of the biodiversity of the prokaryotic complex by depth and by the change in the structure of dominant taxa. Planting a larch forest, which caused acidification of the soil solution and, at the same time, a decrease in organic matter, contributed to a relative increase in acidophilic and acid tolerant microorganisms, as well as oligotrophic microbiota.

## Conclusion

The effect of artificial plantations of larch on the structure of prokaryotic community of mountain-meadow soils of Ai-Petrinskaya Yaila of the Crimean Peninsula was studied. The considered biotopes significantly differ in the structure of the soil microbiome, which was detected both by the relative proportion of microbial taxa and by the analysis of biodiversity. In this case, the factor of the dominant type of vegetation turns out to be leading in relation to the factor of the sampling depth, the effect of which is manifested mainly in the distribution of the richness of individual phylotypes. Changes in the taxonomic structure because of the conversion of the plant community were detected in abundance of high-level taxa in the soil under larch: a significant increase in the proportion and diversity of acidobacteria, bacteria of the phylum *Chloroflexi* and archaea of phylum *Thaumarchaeota*. In the soil under grassland vegetation, the richness of phyla *Verrucomicrobia* and *Proteobacteria* was significantly higher.

Thus, the study demonstrates a significant effect of artificial plantations on the mountain-meadow soil microbiome, which is expressed in the stratification of prokaryotic complex biodiversity by depth and changes in the structure of dominant taxa. The planting of the larch forest, which caused acidification of the soil solution and, at the same time, a decrease in organic matter, contributed to a relative increase in acidophilic and acid-tolerant microorganisms, as well as oligotrophic microbiota.

## Supporting information

S1 Fig Comparison of physic-chemical parameter ranges between soils under grassland vegetation (Grassland) and artificial plantations of larch (Larch). For each chemical parameter boxplot represents statistics for profile values and are normalized to the maximum among both biotopes.
(TIFF)

S2 FigEffect of sampling depth on diversity indices*.Each point on the graph shows the mean and standard error of the mean for the 4 standardized biodiversity indices ("Observed", "Shannon", "Simpson", "PD"). Standardization or z-score normalization ($Z=\frac{X-X}{Sx}$) of biodiversity indices was conducted to be able to generalize them. * This graph shows the form of dependence of biodiversity indices on depth but does not consider the absolute values of the indices in the studied biotopes.(TIFF)

S3 Fig Stress plot for the NMDS-analysis (in Fig 2B). (JPG)

S4 Fig **a. Influence of dominant plant community type and sampling depth on the structure of dominant taxa.** (a) correlations of PC2-axes of dominant taxa (blue) and chemical factors (black) of studied biotopes with depth; (b) values of projections on PC-axes of chemical factors; (c) and abundance of dominant taxa. **b. Statistically significant correlations (p<0.05) of the abundance of some taxa with PC1 (separating biotopes) of physical and chemical factors.** Numbers of taxa correspond to their taxonomic affiliation in S4 Table. **c. Statistically significant correlations (p<0.05) of the abundance of some taxa with PC2 (separating depths) of physical and chemical factors.**
(TIFF)

S1 Table Soil properties under artificial larch plantations and grassland vegetation. The values are presented as Mean±SD.
(DOCX)

S2 Table Values of diversity indices in soil samples under grassland vegetation (Grassland) and artificial plantations of larch (Larch). (DOCX)

S3 Table The relative (percent in the community) abundance of prokaryotic phyla in soils under grassland vegetation (Grassland) and artificial plantations of larch (Larch). The values are presented as ± sd. Roman numerals correspond to the depth of sampling: I—0–5 cm, II—5–10 cm, III—10–15 cm, IV—15–20 cm, V—20–25 cm. Indices of the phyla names: *Aci–Acidobacteria*. *Act–Actinobacteria*, *Bac–Bacteroidetes*, *Chl–Chloroflexi*, *Fir–Firmicutes*, *Gem–Gemmatimonadetes*, *Myx–Mixococcota*, *Pla–Planktomycetes*, *Pro–Proteobacteria*, *Tha–Thaumarchaeota*, *Ver–Verrucomicrobia*.
(DOCX)

S4 Table The average ASV values shown in Fig 4. (DOCX)
