# Supplementary figures and images for: The shifts in the structure of the prokaryotic community of mountain-grassland soil under the influence of artificial larch plantations
Source: PLoS One. 2022 Feb 18;17(2):e0263135. doi: 10.1371/journal.pone.0263135 (PMC8856539; doi:10.1371/journal.pone.0263135)

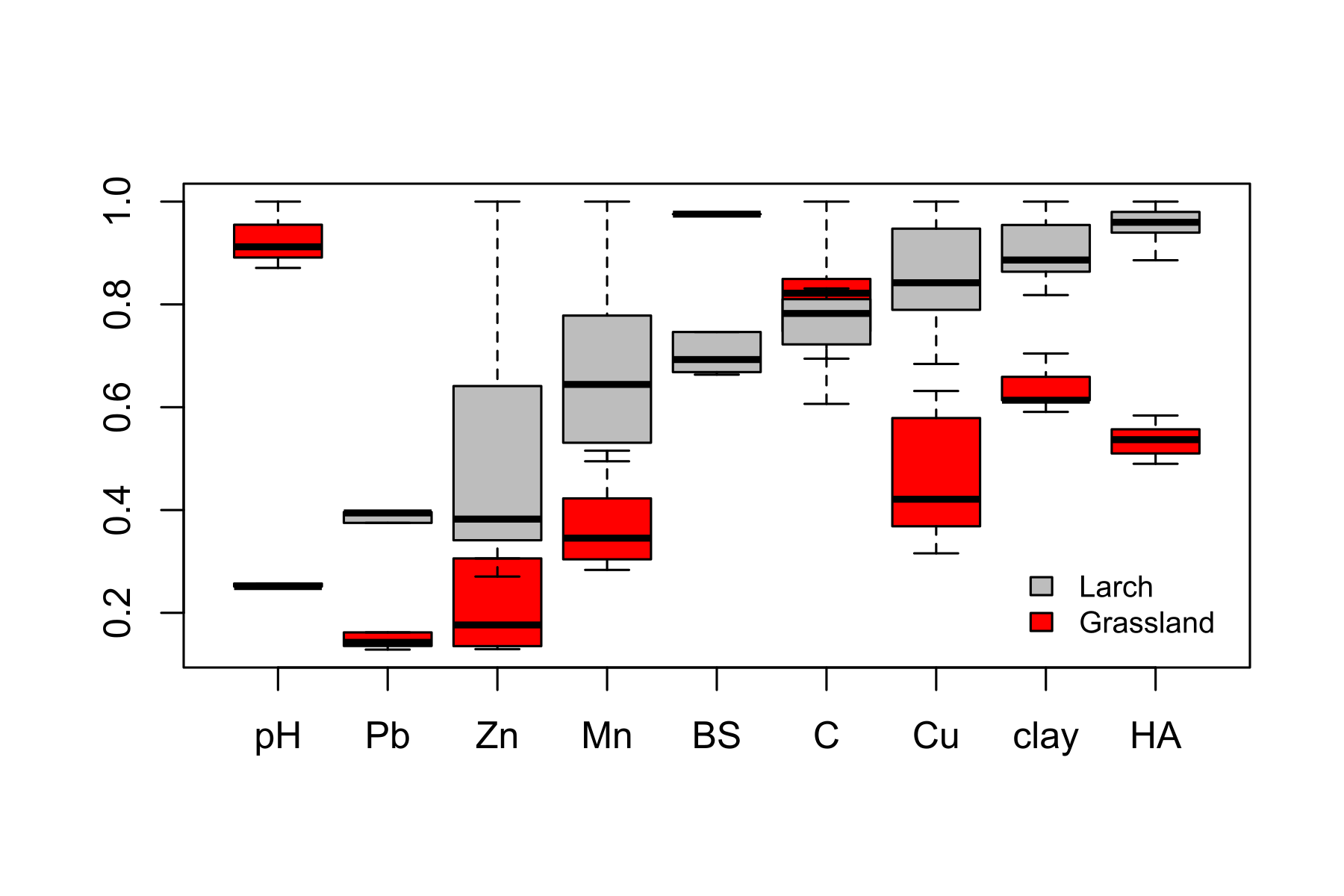

Supplement: S1 Fig — For each chemical parameter boxplot represents statistics for profile values and are normalized to the maximum among both biotopes. (TIFF) [file pone.0263135.s001.tiff]

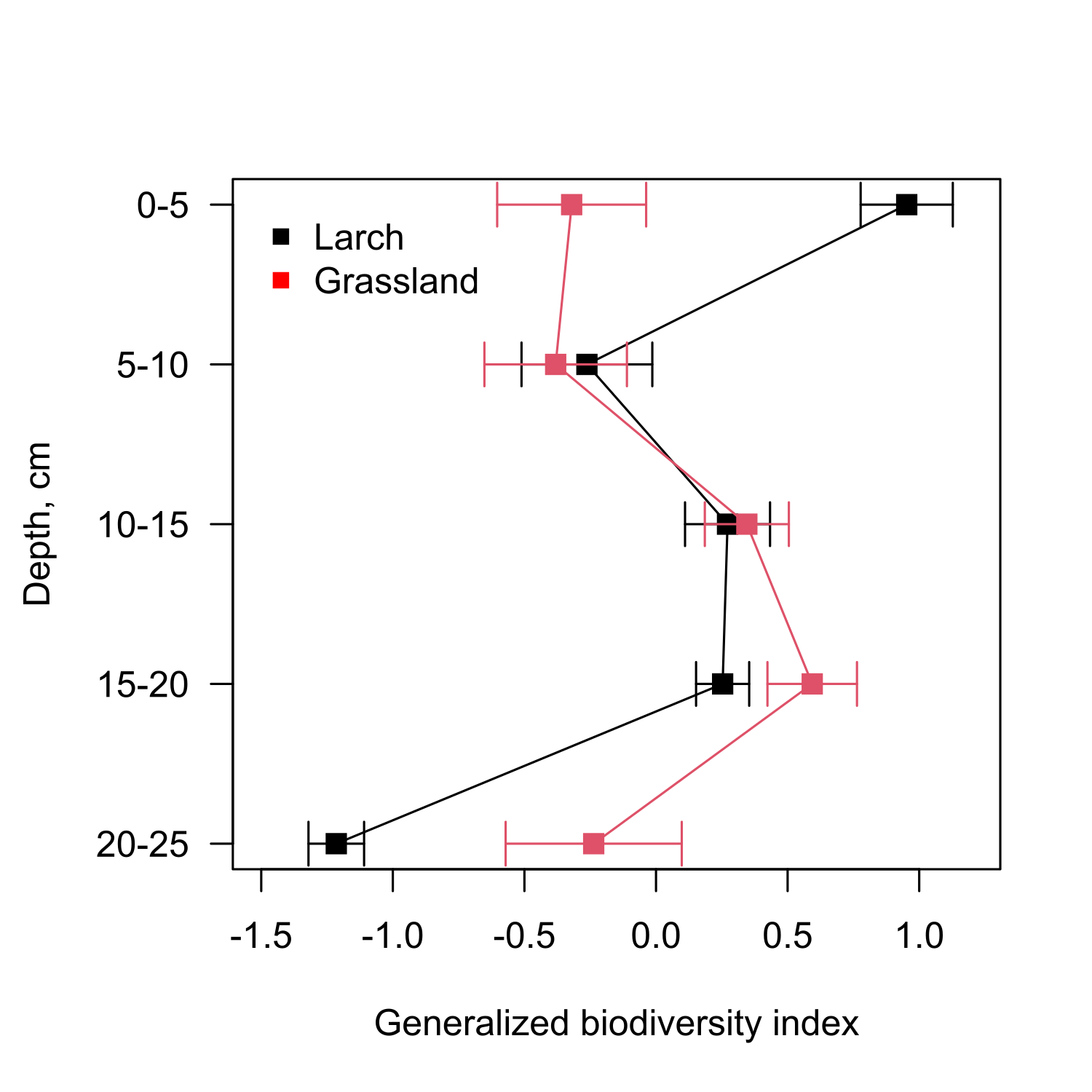

Supplement: S2 Fig — Each point on the graph shows the mean and standard error of the mean for the 4 standardized biodiversity indices ("Observed", "Shannon", "Simpson", "PD"). Standardization or z-score normalization (Z=X−XSx) of biodiversity indices was conducted to be able to generalize them. * This graph shows the form of dependence of biodiversity indices on depth but does not consider the absolute values of the indices in the studied biotopes. (TIFF) [file pone.0263135.s002.tiff]

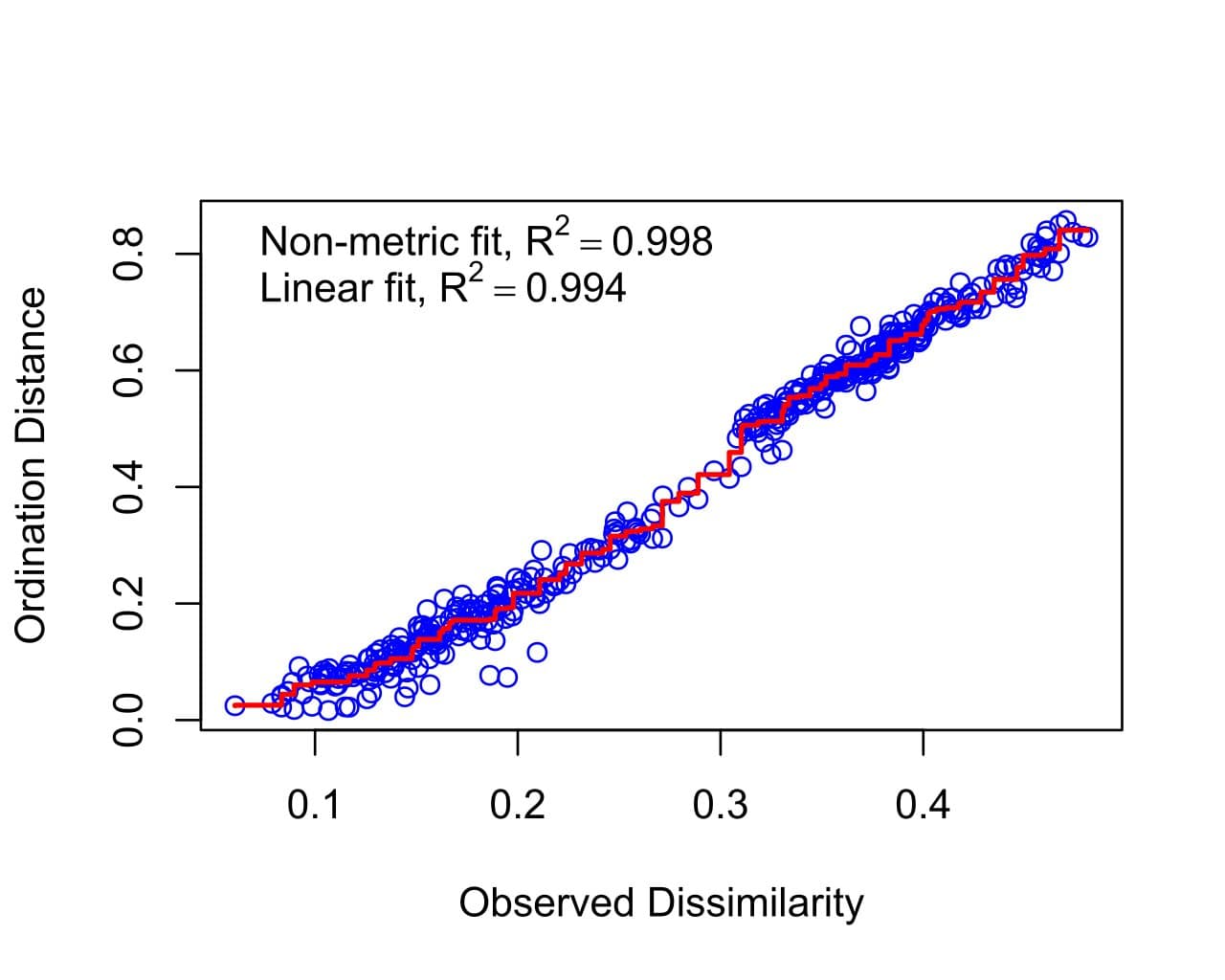

Supplement: S3 Fig — (JPG) [file pone.0263135.s003.jpg]

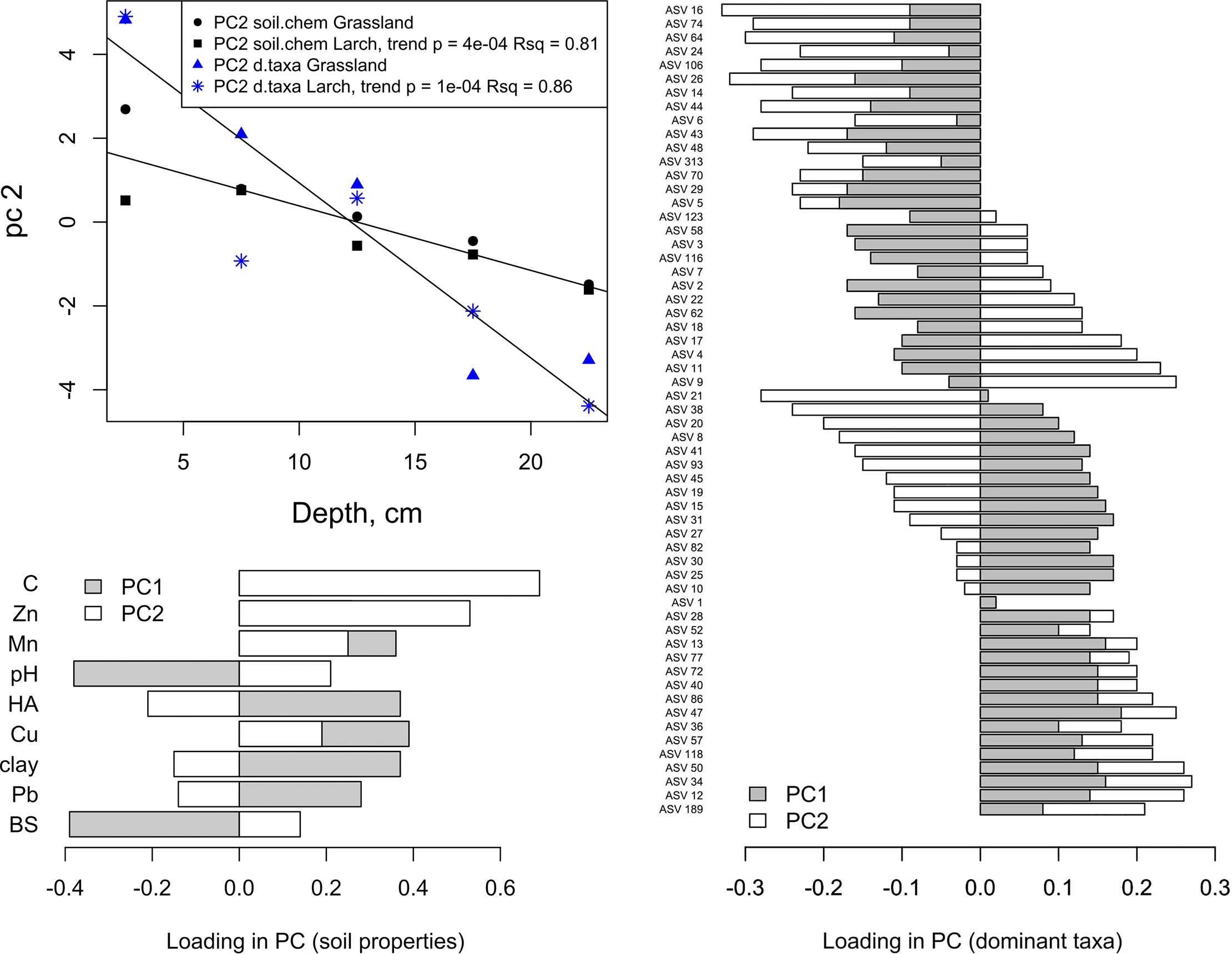

Supplement: S4 Fig — a. Influence of dominant plant community type and sampling depth on the structure of dominant taxa. (a) correlations of PC2-axes of dominant taxa (blue) and chemical factors (black) of studied biotopes with depth; (b) values of projections on PC-axes of chemical factors; (c) and abundance of dominant taxa. b. Statistically significant correlations (p<0.05) of the abundance of some taxa with PC1 (separating biotopes) of physical and chemical factors. Numbers of taxa correspond to their taxonomic affiliation in S4 Table. c. Statistically significant correlations (p<0.05) of the abundance of some taxa with PC2 (separating depths) of physical and chemical factors. (TIFF) [file pone.0263135.s004.tiff]
