# Supplementary material for: The shifts in the structure of the prokaryotic community of mountain-grassland soil under the influence of artificial larch plantations
Source: PLoS One. 2022 Feb 18;17(2):e0263135. doi: 10.1371/journal.pone.0263135 (PMC8856539; doi:10.1371/journal.pone.0263135)
Supplement: S1 Table — The values are presented as Mean±SD. (DOCX) [file pone.0263135.s005.docx]

Supplementary Material: Tables

S1 Table. Soil properties under artificial larch plantations and grassland vegetation. The values are presented as Mean±SD

| **Soil pit** | **Depth, cm** | **Silt, %** | **рН** | **Corg, %** | **HA** | **Base sum (BS)** | **Pb** | **Mn** | **Cu** | **Zn** |
| --- | --- | --- | --- | --- | --- | --- | --- | --- | --- | --- |
|  |  |  |  |  | **cmol(+)*kg^-1^** | | **avialable, mg*kg^-1^** | | | |
| **1378**  **Larch**  **44.475492° N 33.996967° E** | 0-5 | 44±1 | 4.79±0.02 | 3.38±0.18 | 14.3±0.2 | 15.8±0.7 | 2.09±0.06 | 21.3±5.4 | 0.16±0.02 | 1.17±0.11 |
|  | 5-10 | 43±1 | 4.74±0.01 | 3.18±0.06 | 13.1±0.1 | 13.8±0.2 | 1.95±0.41 | 23.2±3.3 | 0.18±0.02 | 1.03±0.06 |
|  | 10-15 | 39±4 | 4.94±0.03 | 3.41±0.11 | 14.0±0.3 | 14.5±0.4 | 5.13±0.78 | 19.0±4.4 | 0.15±0.02 | 0.75±0.13 |
|  | 15-20 | 40±2 | 4.75±0.02 | 3.27±0.10 | 14.5±0.2 | 13.9±0.3 | 1.74±0.11 | 16.1±4.1 | 0.14±0.01 | 0.69±0.15 |
|  | 20-25 | 41±5 | 4.80±0.01 | 2.99±0.02 | 15.0±0.2 | 14.8±0.7 | 2.09±0.23 | 12.7±2.1 | 0.12±0.01 | 0.52±0.08 |
|  | Mean | 41 | 4.80 | 3.25 | 14.2 | 14.2 | 2.60 | 18.5 | 0.15 | 0.83 |
| **1379 Grassland**  **44.475315° N 33.995926° E** | 0-5 | 26±2 | 5.22±0.03 | 4.15±0.15 | 8.7±0.1 | 22.5±4.4 | 1.00±0.24 | 12.9±2.0 | 0.10±0.02 | 1.18±0.10 |
|  | 5-10 | 27±1 | 5.17±0.02 | 3.65±0.03 | 8.4±0.2 | 22.3±3.3 | 0.76±0.23 | 9.8±1.1 | 0.10±0.02 | 0.50±0.04 |
|  | 10-15 | 32±3 | 5.30±0.02 | 3.38±0.15 | 7.2±0.1 | 22.0±2.8 | 0.69±0.17 | 8.7±1.4 | 0.07±0.02 | 0.33±0.04 |
|  | 15-20 | 26±2 | 5.29±0.01 | 3.16±0.10 | 8.0±0.1 | 22.3±3.3 | 0.66±0.16 | 7.9±1.4 | 0.06±0.02 | 0.28±0.06 |
|  | 20-25 | 28±3 | 5.35±0.02 | 2.87±0.22 | 7.7±0.2 | 23.0±3.5 | 0.63±0.15 | 7.4±1.3 | 0.05±0.01 | 0.25±0.04 |
|  | Mean | 28 | 5.26 | 3.44 | 8.0 | 22.4 | 0.79 | 9.3 | 0.08 | 0.51 |
