# Supplementary material for: The shifts in the structure of the prokaryotic community of mountain-grassland soil under the influence of artificial larch plantations
Source: PLoS One. 2022 Feb 18;17(2):e0263135. doi: 10.1371/journal.pone.0263135 (PMC8856539; doi:10.1371/journal.pone.0263135)
Supplement: S2 Table — (DOCX) [file pone.0263135.s006.docx]

Supplementary Material: Tables

S2 Table. Values of diversity indices in soil samples under grassland vegetation (Grassland) and artificial plantations of larch (Larch).

| **Sample (replication)** | **ASV number** | **Shannon index** | **Simpson index** | **Faith’s index (phylogenetic diversity)** |
| --- | --- | --- | --- | --- |
| Grassland.I.1 | 679 | 5.277739 | 0.980154 | 26.61157 |
| Grassland.I.2 | 704 | 5.642952 | 0.990044 | 29.96712 |
| Grassland.I.3 | 669 | 5.08225 | 0.97299 | 26.60684 |
| Grassland.II.1 | 703 | 5.576618 | 0.988757 | 28.92646 |
| Grassland.II.2 | 659 | 5.430761 | 0.985592 | 26.9078 |
| Grassland.II.3 | 573 | 5.548144 | 0.989884 | 27.3776 |
| Grassland.III.1 | 674 | 5.63871 | 0.990232 | 27.57545 |
| Grassland.III.2 | 673 | 5.581435 | 0.989395 | 27.31377 |
| Grassland.III.3 | 754 | 5.691366 | 0.990747 | 30.33287 |
| Grassland.IV.1 | 683 | 5.739508 | 0.993085 | 30.02842 |
| Grassland.IV.2 | 723 | 5.638154 | 0.990609 | 30.71509 |
| Grassland.IV.3 | 678 | 5.616453 | 0.990494 | 27.23139 |
| Grassland.V.1 | 433 | 5.128202 | 0.981763 | 21.15728 |
| Grassland.V.2 | 630 | 5.641444 | 0.991893 | 27.65016 |
| Grassland.V.3 | 721 | 5.723423 | 0.992751 | 30.12928 |
| Lrch.I.1 | 719 | 5.924521 | 0.995134 | 34.11604 |
| Lrch.I.2 | 815 | 6.060052 | 0.995528 | 33.99729 |
| Lrch.I.3 | 801 | 5.9419 | 0.994413 | 35.97277 |
| Lrch.II.1 | 772 | 5.823972 | 0.993646 | 36.99097 |
| Lrch.II.2 | 716 | 5.723718 | 0.991793 | 31.45549 |
| Lrch.II.3 | 681 | 5.600439 | 0.991185 | 28.18261 |
| Lrch.III.1 | 710 | 5.821835 | 0.993712 | 31.53003 |
| Lrch.III.2 | 770 | 5.826141 | 0.99389 | 38.65871 |
| Lrch.III.3 | 756 | 5.897572 | 0.994016 | 32.6209 |
| Lrch.IV.1 | 764 | 5.805356 | 0.993765 | 33.77344 |
| Lrch.IV.2 | 776 | 5.863758 | 0.994274 | 34.69098 |
| Lrch.IV.3 | 715 | 5.7935 | 0.993769 | 30.78323 |
| Lrch.V.1 | 681 | 5.670804 | 0.993505 | 29.48614 |
| Lrch.V.2 | 544 | 5.508413 | 0.990704 | 24.94395 |
| Lrch.V.3 | 617 | 5.594337 | 0.992282 | 28.69095 |
