# Supplementary material for: The shifts in the structure of the prokaryotic community of mountain-grassland soil under the influence of artificial larch plantations
Source: PLoS One. 2022 Feb 18;17(2):e0263135. doi: 10.1371/journal.pone.0263135 (PMC8856539; doi:10.1371/journal.pone.0263135)
Supplement: S3 Table — The values are presented as ± sd. Roman numerals correspond to the depth of sampling: I—0–5 cm, II—5–10 cm, III—10–15 cm, IV—15–20 cm, V—20–25 cm. Indices of the phyla names: Aci–Acidobacteria. Act–Actinobacteria, Bac–Bacteroidetes, Chl–Chloroflexi, Fir–Firmicutes, Gem–Gemmatimonadetes, Myx–Mixococcota, Pla–Planktomycetes, Pro–Proteobacteria, Tha–Thaumarchaeota, Ver–Verrucomicrobia. (DOCX) [file pone.0263135.s007.docx]

Supplementary Material: Tables

S3 Table. The relative (percent in the community) abundance of prokaryotic phyla in soils under grassland vegetation (Grassland) and artificial plantations of larch (Larch). The values are presented as ± sd. Roman numerals correspond to the depth of sampling: I - 0-5 cm, II - 5-10 cm, III - 10-15 cm, IV - 15-20 cm, V - 20-25 cm. Indices of the phyla names: *Aci – Acidobacteria. Act – Actinobacteria, Bac – Bacteroidetes, Chl – Chloroflexi, Fir – Firmicutes, Gem – Gemmatimonadetes, Myx – Mixococcota, Pla – Planktomycetes, Pro – Proteobacteria, Tha – Thaumarchaeota, Ver - Verrucomicrob*

|  | **Grassland** | | | | | **Larch** | | | | |
| --- | --- | --- | --- | --- | --- | --- | --- | --- | --- | --- |
| Phyla | I | II | III | IV | V | I | II | III | IV | V |
| *Aci* | 10,3 ± 2,8 | 13,5 ± 1,6 | 15,8 ± 2,6 | 15,3 ± 1,4 | 15,9 ± 2,3 | 22 ± 1,2 | 25,9 ± 3 | 21,1 ± 1,1 | 22,5 ± 2,5 | 19,4 ± 1,2 |
| *Act* | 12 ± 2,4 | 10,6 ± 0,9 | 11 ± 1,6 | 12,1 ± 1 | 12,4 ± 3 | 11,2 ± 0,8 | 8,1 ± 1,6 | 11,4 ± 1 | 9,2 ± 1,7 | 12,1 ± 0,8 |
| *Bac* | 3,2 ± 2,3 | 5 ± 0,6 | 5,1 ± 0,8 | 3,9 ± 0,9 | 3,9 ± 0,8 | 5,6 ± 0,2 | 2 ± 0,5 | 3,5 ± 1,1 | 4,2 ± 0,2 | 2,2 ± 0 |
| *Chl* | 1,6 ± 0,1 | 2,3 ± 0,3 | 4,1 ± 2,5 | 4,8 ± 0,9 | 4,6 ± 0,8 | 6,8 ± 1 | 9,5 ± 2,1 | 9,6 ± 1,7 | 13 ± 1,7 | 18,6 ± 1,3 |
| *Fir* | 4,6 ± 1,7 | 10,2 ± 0,9 | 9 ± 0,7 | 11,9 ± 2,9 | 8,8 ± 0,9 | 7,6 ± 2,5 | 11,8 ± 1,9 | 10,7 ± 0,8 | 9,5 ± 1,9 | 7,8 ± 1,4 |
| *Gem* | 0,6 ± 0,1 | 1,4 ± 0,4 | 1,3 ± 0,3 | 1,4 ± 0,2 | 1,6 ± 0,7 | 2 ± 0,3 | 2,5 ± 0,7 | 2,4 ± 0,4 | 2 ± 0,4 | 1,7 ± 0,5 |
| *Myx* | 1,3 ± 0,3 | 1,1 ± 0,4 | 1,4 ± 0,5 | 1,5 ± 0,5 | 1,5 ± 0,3 | 1,2 ± 0,2 | 0,6 ± 0,1 | 0,9 ± 0,1 | 0,8 ± 0,3 | 0,7 ± 0,1 |
| *Pla* | 2,7 ± 0,8 | 3,5 ± 0,7 | 4,5 ± 0,9 | 4,9 ± 0,2 | 4,8 ± 0,8 | 2,3 ± 0,3 | 1,9 ± 0,3 | 2,5 ± 0,5 | 2,1 ± 0,1 | 1,8 ± 0,4 |
| *Pro* | 17,1 ± 2,4 | 15,2 ± 0,6 | 13,9 ± 0,8 | 14,9 ± 1,8 | 11,9 ± 3 | 24,8 ± 3,9 | 16,5 ± 1 | 18 ± 1,2 | 17,6 ± 1,3 | 14 ± 1,6 |
| *Tha* | 0,4 ± 0,3 | 0,4 ± 0,2 | 0,3 ± 0,1 | 0,4 ± 0,1 | 0,2 ± 0,1 | 1,2 ± 0,2 | 1,4 ± 1,1 | 2,4 ± 0,8 | 2,3 ± 0,4 | 2,1 ± 0,9 |
| *Ver* | 44 ± 12,7 | 33,4 ± 5,3 | 30,6 ± 1,3 | 25,6 ± 1,4 | 31 ± 11,3 | 11,8 ± 0,4 | 17 ± 2,6 | 14,4 ± 0,7 | 13,3 ± 1,7 | 16 ± 0,6 |
