# Supplementary material for: The shifts in the structure of the prokaryotic community of mountain-grassland soil under the influence of artificial larch plantations
Source: PLoS One. 2022 Feb 18;17(2):e0263135. doi: 10.1371/journal.pone.0263135 (PMC8856539; doi:10.1371/journal.pone.0263135)
Supplement: S4 Table — (DOCX) [file pone.0263135.s008.docx]

Supplementary Material: Tables

S4 Table. The average ASV values shown in Figure 3.

| **ASV**  **ID** | **Larch** | | **Grassland** | | | **phyla** | **class** | **order** | **family** | **genus** |
| --- | --- | --- | --- | --- | --- | --- | --- | --- | --- | --- |
| 40 | | 0.8 | | 0 | Proteobacteria | | Gammaproteobacteria | WD260 | NA | NA |
| 30 | | 0.8 | | 0 | Crenarchaeota | | Nitrososphaeria | Nitrososphaerales | Nitrososphaeraceae | NA |
| 58 | | 0.1 | | 0.8 | Verrucomicrobiota | | Verrucomicrobiae | Chthoniobacterales | Chthoniobacteraceae | Candidatus_Udaeobacter |
| 5 | | 0.1 | | 0.8 | Verrucomicrobiota | | Verrucomicrobiae | Chthoniobacterales | Chthoniobacteraceae | Candidatus_Udaeobacter |
| 47 | | 0.8 | | 0.1 | Proteobacteria | | Alphaproteobacteria | Elsterales | NA | NA |
| 25 | | 0.9 | | 0.2 | Acidobacteriota | | Acidobacteriae | Acidobacteriales | NA | NA |
| 62 | | 0 | | 0.6 | Verrucomicrobiota | | Verrucomicrobiae | Chthoniobacterales | Xiphinematobacteraceae | Candidatus_Xiphinematobacter |
| 116 | | 0 | | 0.6 | Proteobacteria | | Gammaproteobacteria | Burkholderiales | SC-I-84 | NA |
| 29 | | 0.1 | | 0.7 | Planctomycetota | | Phycisphaerae | Tepidisphaerales | WD2101_soil_group | NA |
| 43 | | 0.1 | | 0.7 | Proteobacteria | | Alphaproteobacteria | Rhizobiales | Xanthobacteraceae | NA |
| 36 | | 0.6 | | 0 | Acidobacteriota | | Acidobacteriae | Acidobacteriales | NA | NA |
| 15 | | 0.6 | | 0 | Chloroflexi | | Ktedonobacteria | Ktedonobacterales | Ktedonobacteraceae | HSB_OF53-F07 |
| 72 | | 0.6 | | 0 | Chloroflexi | | Ktedonobacteria | Ktedonobacterales | Ktedonobacteraceae | NA |
| 77 | | 0.6 | | 0 | Chloroflexi | | Ktedonobacteria | Ktedonobacterales | Ktedonobacteraceae | Ktedonobacter |
| 31 | | 0.6 | | 0 | Chloroflexi | | Ktedonobacteria | Ktedonobacterales | Ktedonobacteraceae | HSB_OF53-F07 |
| 27 | | 0.7 | | 0.1 | Proteobacteria | | Gammaproteobacteria | Burkholderiales | Oxalobacteraceae | Glaciimonas |
| 123 | | 0 | | 0.5 | Proteobacteria | | Gammaproteobacteria | Burkholderiales | Rhodocyclaceae | Georgfuchsia |
| 70 | | 0.1 | | 0.6 | Verrucomicrobiota | | Verrucomicrobiae | Chthoniobacterales | Chthoniobacteraceae | Candidatus_Udaeobacter |
| 2 | | 0.1 | | 0.6 | Verrucomicrobiota | | Verrucomicrobiae | Chthoniobacterales | Chthoniobacteraceae | Candidatus_Udaeobacter |
| 57 | | 0.5 | | 0 | Acidobacteriota | | Acidobacteriae | Acidobacteriales | NA | NA |
| 86 | | 0.5 | | 0 | Proteobacteria | | Gammaproteobacteria | WD260 | NA | NA |
| 45 | | 0.5 | | 0 | Crenarchaeota | | Nitrososphaeria | Nitrososphaerales | Nitrososphaeraceae | NA |
| 34 | | 0.6 | | 0.1 | Acidobacteriota | | Acidobacteriae | Bryobacterales | Bryobacteraceae | Bryobacter |
| 50 | | 0.6 | | 0.1 | Acidobacteriota | | Acidobacteriae | Acidobacteriales | NA | NA |
| 19 | | 0.6 | | 0.1 | Verrucomicrobiota | | Verrucomicrobiae | Chthoniobacterales | Chthoniobacteraceae | Candidatus_Udaeobacter |
| 13 | | 0.8 | | 0.3 | Verrucomicrobiota | | Verrucomicrobiae | Chthoniobacterales | Chthoniobacteraceae | Candidatus_Udaeobacter |
| 28 | | 0.8 | | 0.3 | Acidobacteriota | | Acidobacteriae | Subgroup_2 | NA | NA |
| 26 | | 0.2 | | 0.7 | Acidobacteriota | | Blastocatellia | Pyrinomonadales | Pyrinomonadaceae | RB41 |
| 44 | | 0.2 | | 0.7 | Bacteroidota | | Bacteroidia | Chitinophagales | Chitinophagaceae | Terrimonas |
| 3 | | 0.2 | | 0.7 | Verrucomicrobiota | | Verrucomicrobiae | Chthoniobacterales | Chthoniobacteraceae | Candidatus_Udaeobacter |
| 64 | | 0 | | 0.4 | Planctomycetota | | Phycisphaerae | Tepidisphaerales | WD2101_soil_group | NA |
| 106 | | 0 | | 0.4 | Acidobacteriota | | Blastocatellia | Pyrinomonadales | Pyrinomonadaceae | RB41 |
| 74 | | 0.1 | | 0.5 | Gemmatimonadota | | Gemmatimonadetes | Gemmatimonadales | Gemmatimonadaceae | NA |
| 93 | | 0.4 | | 0 | Chloroflexi | | Ktedonobacteria | Ktedonobacterales | Ktedonobacteraceae | HSB_OF53-F07 |
| 82 | | 0.4 | | 0 | Bacteroidota | | Bacteroidia | Chitinophagales | Chitinophagaceae | Puia |
| 52 | | 0.4 | | 0 | Chloroflexi | | Ktedonobacteria | Ktedonobacterales | Ktedonobacteraceae | NA |
| 10 | | 0.8 | | 0.4 | Firmicutes | | Bacilli | Bacillales | Planococcaceae | Paenisporosarcina |
| 41 | | 0.6 | | 0.2 | Acidobacteriota | | Acidobacteriae | Acidobacteriales | NA | NA |
| 12 | | 0.7 | | 0.3 | Proteobacteria | | Alphaproteobacteria | Rhizobiales | Xanthobacteraceae | Bradyrhizobium |
| 48 | | 0.1 | | 0.4 | Proteobacteria | | Gammaproteobacteria | Burkholderiales | Oxalobacteraceae | Massilia |
| 118 | | 0.4 | | 0.1 | Actinobacteriota | | Thermoleophilia | Gaiellales | NA | NA |
| 8 | | 0.4 | | 0.1 | Chloroflexi | | Ktedonobacteria | Ktedonobacterales | Ktedonobacteraceae | HSB_OF53-F07 |
| 313 | | 0 | | 0.3 | Proteobacteria | | Gammaproteobacteria | Burkholderiales | Oxalobacteraceae | Undibacterium |
| 11 | | 0.2 | | 0.5 | Proteobacteria | | Alphaproteobacteria | Rhizobiales | Xanthobacteraceae | NA |
| 189 | | 0.3 | | 0 | Proteobacteria | | Gammaproteobacteria | Burkholderiales | TRA3-20 | NA |
| 16 | | 0.3 | | 0.6 | Verrucomicrobiota | | Verrucomicrobiae | Chthoniobacterales | Chthoniobacteraceae | Candidatus_Udaeobacter |
| 7 | | 0.4 | | 0.7 | Bacteroidota | | Bacteroidia | Chitinophagales | Chitinophagaceae | Puia |
| 14 | | 0.6 | | 0.8 | Verrucomicrobiota | | Verrucomicrobiae | Chthoniobacterales | Chthoniobacteraceae | Candidatus_Udaeobacter |
| 38 | | 0.4 | | 0.2 | Chloroflexi | | Ktedonobacteria | Ktedonobacterales | Ktedonobacteraceae | HSB_OF53-F07 |
| 4 | | 0.4 | | 0.6 | Verrucomicrobiota | | Verrucomicrobiae | Chthoniobacterales | Chthoniobacteraceae | Candidatus_Udaeobacter |
| 22 | | 0.4 | | 0.6 | Verrucomicrobiota | | Verrucomicrobiae | Chthoniobacterales | Chthoniobacteraceae | Candidatus_Udaeobacter |
| 17 | | 0.4 | | 0.6 | Proteobacteria | | Alphaproteobacteria | Rhizobiales | Xanthobacteraceae | NA |
| 18 | | 0.4 | | 0.6 | Verrucomicrobiota | | Verrucomicrobiae | Chthoniobacterales | Chthoniobacteraceae | Candidatus_Udaeobacter |
| 20 | | 0.6 | | 0.4 | Acidobacteriota | | Acidobacteriae | Subgroup_2 | NA | NA |
| 9 | | 0.7 | | 0.8 | Proteobacteria | | Alphaproteobacteria | Rhizobiales | Xanthobacteraceae | Bradyrhizobium |
| 6 | | 0.7 | | 0.8 | Firmicutes | | Bacilli | Bacillales | NA | NA |
| 24 | | 0.4 | | 0.5 | Acidobacteriota | | Acidobacteriae | Subgroup_2 | NA | NA |
| 21 | | 0.4 | | 0.4 | Actinobacteriota | | Thermoleophilia | Gaiellales | NA | NA |
| 1 | | 0.7 | | 0.7 | Firmicutes | | Bacilli | Bacillales | NA | NA |
